# Data on identification of conserved and novel miRNAs in *Elettaria cardamomum*

**DOI:** 10.1016/j.dib.2017.08.037

**Published:** 2017-09-05

**Authors:** F. Nadiya, N. Anjali, Jinu Thomas, A. Gangaprasad, K.K. Sabu

**Affiliations:** a Biotechnology and Bioinformatics Division, Jawaharlal Nehru Tropical Botanic Garden and Research Institute (JNTBGRI), Palode, Thiruvananthapuram 695562, India; b Department of Botany, University of Kerala, Thiruvananthapuram 695581, India

**Keywords:** miRNA, Cardamom, Cultivar, Spice crop, Wild genotype

## Abstract

*Elettaria cardamomum* (L.) Maton, or small cardamom referred as ‘queen of spices’, is a perennial herbaceous rhizomatous monocot of the family Zingiberaceae. Cardamom seeds and fruits are the economically significant parts and effectively used as a traditional medicine, food additive and flavoring agent. In the present study, using Ion Proton next generation sequencing technology we performed the small RNA sequencing, conserved and novel miRNA predictions of a wild and five cultivar genotypes of cardamom. Small RNA sequencing generated a total of 5,451,328 and 2,756,250 raw reads for wild and cultivar cardamom respectively. The raw data was submitted to SRA database of NCBI under the accession numbers and SRX2273863 (wild) and SRX2273862 (cultivars). The raw reads were quality filtered and predicted conserved and novel miRNAs for wild and cultivar cardamom. The predicted miRNAs, miRNA-targets and functional annotations might provide valuable insights into differences between wild progenitor and cultivated cardamom.

Specifications Table:

**Table:** 

Subject area	Biology
More specific subject area	Plant molecular biology
Type of data	Small RNA sequence data
How data was acquired	Next generation sequencing using Ion Proton System^(^^TM)^
Data format	Raw data in FASTQ file
Experimental factors	Small RNA sequencing of tissues of wild and cultivar cardamom
Experimental features	Freshly collected leaf, stem, flower, flower buds and young fruits were used for RNA isolation, tissues were pooled, Small RNA seq libraries representing two genotypes of cardamom were sequenced and predicted known and novel miRNAs
Data source location	JNTBGRI, cardamom germplasm conservatory
Data accessibility	NCBI, SRA

Value of the data

• The data presented in the article provides information on genome wide conserved and novel miRNA profiling and differential expression analysis of wild and cultivar genotypes of cardamom.
• This data identifies miRNAs participating in the negative regulation of significant transcription factors, enzymes and other mRNAs involved in plant development, stress responses and pathogenic resistance.
• The identified miRNAs were also compared with other monocots and *Arabidopsis* to determine the extent of conservation of known miRNAs between cardamom, other monocots and *Arabidopsis*.
• The data allows other researchers to study miRNA regulations in various biosynthetic pathways.

## 1 Data

The data shared in this article provides information on conserved and novel miRNAs and differential miRNA expression analysis between wild and cultivar genotypes of cardamom.

https://www.ncbi.nlm.nih.gov/sra/SRX2273862[accn].

https://www.ncbi.nlm.nih.gov/sra/SRX2273863[accn].

## 2 Experimental design, materials and methods

### 2.1 Plant material

Fresh leaves, stem, flower, flower buds and young fruits were collected from wild and five cultivar (including landraces and released varieties) accessions of cardamom growing in the JNTBGRI cardamom conservatory. The tissues were immediately frozen in liquid nitrogen and kept as such until processing.

### 2.2 Total RNA isolation and small RNA sequencing

Total RNA was isolated from leaves, stem, flower, flower buds and young fruits of cardamom using miRNeasy mini kit combined with modified CTAB method [1]. Quantification and quality analysis were done using 2100 BioAnalyzer (Agilent technologies). The tissues of wild cardamom were pooled as one sample and the tissues from all cultivars were pooled as second sample representing wild and cultivar cardamom. Two sets of total RNA enriched with miRNAs were purified and cDNA library was constructed using Ion total RNA seq Kit V2 according to manufacturer's instructions. The purified libraries were submitted to small RNA sequencing with the Ion Proton Sequencer.

### 2.3 Identification of conserved and novel miRNAs

The raw reads were preprocessed to remove adapter sequences, rRNAs, tRNAs, other small RNAs and sequences below 17 and 27nt. The identical sequences were collapsed and known miRNAs were identified by performing blastn search against plant mature miRNAs from miRBase database. The remaining reads were mapped to the *Curcuma longa* (Zingiberaceae family) transcriptome sequence (the whole genome or transcriptome sequence of cardamom was not available at the time of the study) to predict putative novel miRNAs using miRDeep-P software [2] satisfying Mayer's criteria [3] of miRNA prediction (Table 1). Using the identified miRNAs we also performed the differential expression analysis, target prediction and Gene Ontology (GO) annotations for wild and cultivar genotypes of cardamom. The information we provided might furnish awareness on genetic resources of wild cardamom, molecular marker development and breeding for developing novel elite cultivars using RNAi technology. The study also provides insight into the genetic variation in wild and domesticated cardamom.

**Table 1:** Read and assembly statistics.

Plant material	Wild	Cultivar
Raw reads obtained after sequencing	5,451,328	2,756,250
Reads after adapter removal	5,375,277 (98.6%)	2,720,430 (98.7%)
Reads after other RNAs removed	3,524,114 (64.6%)	1,794,905 (65.1%)
Reads after size filtering	2,066,216 (37.9%)	1,125,109 (40.8%)
Unique sequences obtained after collapsing	1,080,645 (19.8%)	588,044 (21.3%)
Number of reads aligned to miRBase database	1559	1368
Number of known miRNAs	138	141
Reads retained for novel miRNA prediction	1,015,420 (18.6%)	547,739 (19.8%)
Number of reads aligned to *Curcuma longa*	487,401	277,428
Novel miRNAs predicted	9	5

### 2.4 Differential expression analysis and target prediction

Count of each miRNA was normalized to Tags per million (TPM) to compare the abundance of miRNAs in wild and cultivar library. Fold change of each miRNA between the two libraries was calculated employing the formula: log_2_ (cultivars/wild) [4]. Unique potential targets were predicted for both novel and conserved miRNAs using psRNA target [5].
